# A rapid, non-invasive procedure for quantitative assessment of drought survival using chlorophyll fluorescence

**DOI:** 10.1186/1746-4811-4-27

**Published:** 2008-11-11

**Authors:** Nick S Woo, Murray R Badger, Barry J Pogson

**Affiliations:** 1 Australian Research Council Centre of Excellence in Plant Energy Biology, School of Biochemistry and Molecular Biology, the Australian National University, Canberra, ACT 0200, Australia; 2 Australian Research Council Centre of Excellence in Plant Energy Biology, Research School of Biological Sciences, the Australian National University, Canberra, ACT 0200, Australia

## Abstract

**Background:**

Analysis of survival is commonly used as a means of comparing the performance of plant lines under drought. However, the assessment of plant water status during such studies typically involves detachment to estimate water shock, imprecise methods of estimation or invasive measurements such as osmotic adjustment that influence or annul further evaluation of a specimen's response to drought.

**Results:**

This article presents a procedure for rapid, inexpensive and non-invasive assessment of the survival of soil-grown plants during drought treatment. The changes in major photosynthetic parameters during increasing water deficit were monitored via chlorophyll fluorescence imaging and the selection of the maximum efficiency of photosystem II (F_v_/F_m_) parameter as the most straightforward and practical means of monitoring survival is described. The veracity of this technique is validated through application to a variety of *Arabidopsis thaliana *ecotypes and mutant lines with altered tolerance to drought or reduced photosynthetic efficiencies.

**Conclusion:**

The method presented here allows the acquisition of quantitative numerical estimates of *Arabidopsis *drought survival times that are amenable to statistical analysis. Furthermore, the required measurements can be obtained quickly and non-invasively using inexpensive equipment and with minimal expertise in chlorophyll fluorometry. This technique enables the rapid assessment and comparison of the relative viability of germplasm during drought, and may complement detailed physiological and water relations studies.

## Background

With the increasing demands of industrial, municipal and agricultural consumption on dwindling water supplies [1], the development of sustainable farming practices has taken higher priority. For this reason, advancement of the current understanding of plant responses to drought stress and the mechanisms involved has become a major target of research and investment, with the ultimate goal of developing crops with improved water use efficiencies and minimized drought-induced loss of yield [2,3]. On a multi-gene scale, analysis of quantitative trait loci allows identification of genetic regions responsible for control of complex responses such as the co-ordination of the whole-plant response to water deficit [4,5]. In parallel to this, as our comprehension of the molecular signaling events leading to drought responses has increased, genetic engineering techniques now also permit the manipulation of these response mechanisms through targeted overexpression or suppression of specific genes [3,6].

Irrespective of the method used to generate plants with altered drought responses, their performance under drought conditions must be evaluated in order to determine their effectiveness. This introduces a number of experimental decisions, not only with respect to the manner in which water deficit is applied, but also the means used to assess the drought stress response. In regards to the application of water deficit to small model plants such as *Arabidopsis thaliana *several alternative procedures are in common use, including the detachment of leaves or whole rosettes [7], air-drying of uprooted plants [8], or the transfer of specimens to solute-infused media [9]. Rosette detachment and uprooting are suitable for assessment of a plant's ability to resist rapid water loss using dehydration avoidance mechanisms, such as stomatal closure. In contrast, growth on solute-infused media allows exposure of specimens to a defined level of water deficit over a longer period of time, and thus is a valid means of evaluating adaptive responses [10]. Possibly the most straightforward and relevant application of drought stress is through experiments where water is withheld from soil-grown plants. Soil-drying techniques are generally regarded as the most practical means of approximating field drought conditions for laboratory-based research. However, their use introduces complicating factors such as variation in leaf or soil water loss rates due to differences in plant size and soil composition [10,11] and may necessitate the monitoring and adjustment or control of soil water content [12,13].

In order for soil-drying experiments to yield quantifiable comparisons between genotypes it is crucial that a suitable method of assessment be employed [11,14]. Measurements of stomatal conductance [15,16], leaf or soil water potential [12,17] or plant relative water content (RWC) [12] provide meaningful quantitative data and are necessary in a detailed physiological analysis of drought response characteristics. However, determination of leaf water potential or water content involves destructive analyses that may influence future measurements and may not accurately represent the plant as a whole. Physical disturbance to specimens is also typically unavoidable during analyses of transpiration and soil water content. The simplest assessment of viability in response to drought is the capacity of a plant to grow and remain alive under progressively increasing water deficit conditions, and thus it is common practice to utilize such survival assays to compare the drought performance of different plant lines. In such survival experiments, watering is resumed after the majority of specimens appear to have perished, and the percentage of surviving (viable) plants is presented as a measure of the drought tolerance of a line [7,18-20]. However, these survival studies rely on qualitative observation of physical symptoms of water deficit stress such as turgor loss, chlorosis, and other qualities that can vary greatly between specimens and are also sensitive to experimental conditions. Critically, the timing of rehydration presents a major problem; for instance, for plants that fail to recover upon rewatering, it is not be possible to determine retrospectively the time at which they perished. Thus, current laboratory-based techniques require either invasive or destructive measurements or are largely subjective and qualitative.

With respect to drought, the negative impact on photosynthesis is well-documented, with carbon assimilation declining progressively with increasing water deficit as a result of both stomatal and metabolic limitations [21-24]. Thus, non-invasive measurement of photosynthesis by chlorophyll *a *fluorometry [25,26] may potentially provide a means to determine plant viability and performance in response to drought. Measurement of chlorophyll fluorescence by probe-based systems has been utilized for non-invasive analyses of stress-induced perturbations to photosynthesis for several decades [27,28]. Indeed, dissection and analysis of the rapid polyphasic chlorophyll *a *fluorescence transient OJIP [29], a technique applied previously to measure tolerance to light [30] and chilling [31] stresses, was recently employed to assess the response of several barley cultivars to non-lethal drought stress [32]. The recent introduction of chlorophyll fluorescence imaging systems has allowed acquisition of fluorescence data from larger sample areas than probe-based systems [33,34], thereby enabling simultaneous measurement of several specimens and the identification of spatial heterogeneities in photosynthesis across whole leaves or rosettes. Such imaging techniques have also been successfully utilized to examine the impact of numerous environmental stresses [35], including cold [36,37], high light [38] and wounding [34].

In this article, we tested the response of major photosynthetic parameters to increasing water deficit in *Arabidopsis *with the objective of developing a rapid, reproducible, accurate and non-invasive method for monitoring plant viability in response to prolonged drought. We have developed a procedure that allows a quantitative and precise determination of viability in intact, drought-stressed *Arabidopsis *plants. The accuracy and general application of this technique has been demonstrated in different wild-type cultivars and in mutant lines that possess differences in drought performance or altered photosynthetic characteristics.

## Results

### Identification of drought-induced changes in photosynthetic parameters in *Arabidopsis *wild-type ecotypes

In order to identify a parameter suitable for monitoring survival in *Arabidopsis *in response to water deficit, an assessment of common photosynthetic parameters was performed spanning the duration of a prolonged, terminal drought treatment. To verify that any observed trends would be applicable across experiments involving *Arabidopsis *lines of different ecotypic backgrounds, three commonly-used species of *Arabidopsis *were examined: Columbia (Col), Landsberg *erecta *(L*er*) and C24.

**Figure 1:**
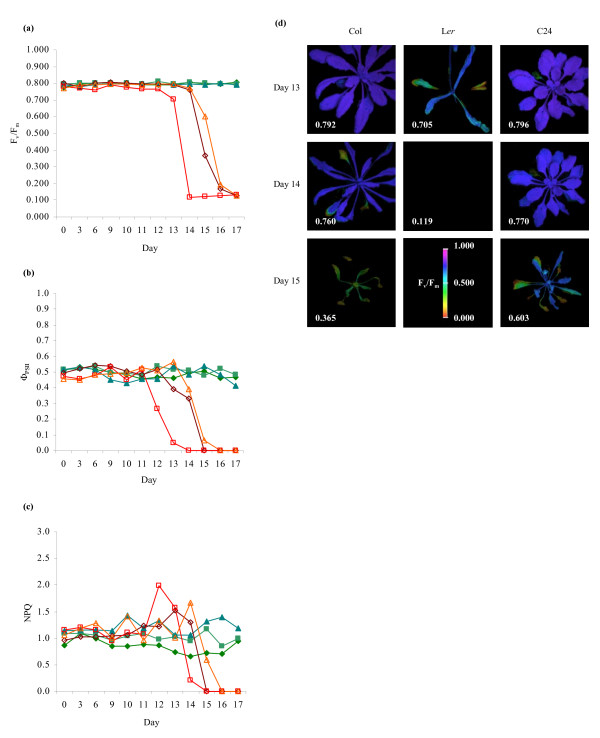
**Measurements of (a) F_v_/F_m_, (b) Φ_PSII _and (c) NPQ during progression of drought.** Measurements are shown for Columbia (◇), Landsberg (□) and C24 (Δ) plants; filled symbols represent controls, empty symbols represent drought-treated plants. For both control and drought-treated populations, *n *= 8 for each line; for clarity, only measurements from one control and one drought specimen of each line are displayed (see Additional file 1b for additional F_v_/F_m _data). (d) False-colour images of F_v_/F_m _measurements obtained from drought-affected specimens during late drought. The average F_v_/F_m _measurements of each plant are shown in the lower left corner of the respective images. Note that false-colour images were not generated at F_v_/F_m _values of < ~0.125; for details, refer to Experimental Procedures. The same individual specimens provided all the measurements presented in Figure 1a-d.

The maximum efficiency of photosystem II (F_v_/F_m_) and operating efficiency of photosystem II (Φ_PSII_) represent the capacity for photon energy absorbed by photosystem II (PSII) to be utilized in photochemistry under dark- and light-adapted conditions respectively [25,39]. As shown in Figures 1a and 1d, F_v_/F_m _did not vary from levels expected for plants under non-stressed conditions (~0.800) until late in the course of the treatment, when a slight decline (to 0.700–0.750) was observed. This was followed by a sudden and rapid decline to very low levels (0.100–0.250) over a 2–3-day period, after which very little change was noted. This decrease in F_v_/F_m _affected all rosette leaves and was readily discernible from false-colour images of F_v_/F_m _measurements (Figure 1d). For clarity, Figure 1a shows representative measurements from a single plant of each ecotype; refer to Additional file 1b for data from additional biological replicates. Φ_PSII _levels under the growth illumination conditions were likewise stable until the latter stages of drought, at which time a rapid decline was observed (Figure 1b). This decline appeared to precede the decline in F_v_/F_m _by approximately one day; often Φ_PSII _fell to 50% or less of normal levels before an appreciable change in F_v_/F_m _was noted (Additional file 1a).

Under conditions where absorption of photons exceeds the capacity for their utilization in photochemical processes, excess excitation energy may be dissipated as thermal radiation via xanthophyll-mediated non-photochemical quenching (NPQ) [40]. NPQ did not show appreciable changes for most of the treatment, with values ranging from approximately 0.8–1.6 (Figure 1c). During late drought, NPQ levels tended towards the higher end of this range, around 1.6–1.8. This slight increase was followed by a more pronounced decrease to minimal levels, and eventually nil. A number of other photosynthetic parameters were also monitored, including the rate of photosynthetic electron transport (ETR) (Additional file 1c) [39] and non-regulated energy dissipation (Φ_NO_) (Additional file 1d) [41]. The chlorophyll fluorescence measurements from which the above photosynthetic parameters have been derived are provided in Additional file 2. All parameters investigated underwent similar changes to those described above, remaining mostly constant before undergoing a sudden, catastrophic decline (or, in the case of Φ_NO_, a sudden increase) to critical levels. The rapid decline in photosynthetic parameters occurred concurrently with the appearance of physical symptoms of drought stress, including chlorosis of leaves and loss of turgor (Figure 1d). As F_v_/F_m _is the most readily measurable of these parameters, it was investigated further.

### Correlation of the decline in F_v_/F_m _with decreased plant water status and viability

**Figure 2:**
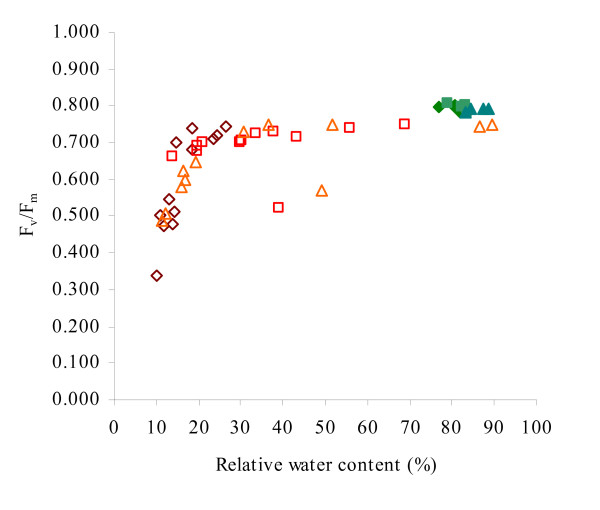
**Relationship between F_v_/F_m _and plant relative water content.** Measurements are shown for Columbia (◇), Landsberg (□) and C24 (Δ) plants; filled symbols represent controls, empty symbols represent drought-treated plants. For control populations, *n *= 4 for each line; for drought-treated populations, *n *= 12 for each line. Data shown are representative of two separate experiments.

To determine if the rapid decline in F_v_/F_m _during late drought correlates with deterioration in plant water status, the RWC of drought-affected plants exhibiting signs of photosynthetic decline (F_v_/F_m _< 0.750) was determined (Figure 2). Well-watered plants had RWCs of 80–90% and F_v_/F_m _levels of ~0.800. Under drought conditions, for RWCs in the range of 20–80%, F_v_/F_m _varied between 0.700–0.750. Plants experiencing critical levels of water deficiency (RWC of 10–20%) displayed noticeably depressed F_v_/F_m _levels, in the range of 0.450–0.750. The close correlation between the sudden decline in F_v_/F_m _and critical levels of water deficit suggest that the rapid changes in F_v_/F_m _may be a useful indicator of terminal water loss, or loss of viability, at which point plants are unable to recover even if the soil is rehydrated. Association of this loss of viability with the decline of F_v_/F_m _beyond a 'threshold' value would provide a convenient, non-invasive means of identifying the time of death of plants subjected to drought.

**Figure 3:**
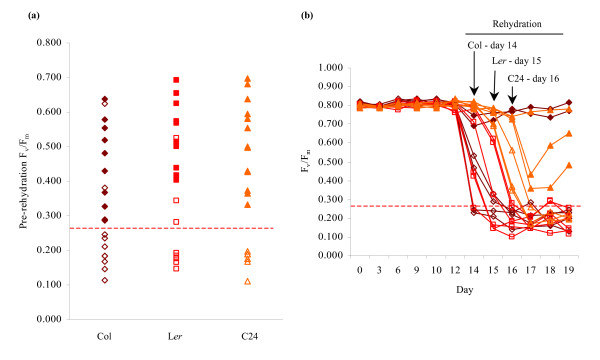
**Validation of the F_v_/F_m _threshold test for viability.** Drought-affected Columbia (◇), Landsberg (□) and C24 (Δ) plants were rehydrated after their F_v_/F_m _levels were observed to fall below 0.750. Filled symbols represent plants that recovered within 3 days of rehydration, while empty symbols represent plants that failed to evidence signs of recovery following watering. The 33% threshold for a typical average control F_v_/F_m _of 0.800 is shown as a dotted line. (a) F_v_/F_m _measurements of individual specimens immediately prior to rehydration. For each line, *n *= 20. (b) Change in F_v_/F_m _of drought-treated plants following rehydration. Columbia, Landsberg and C24 plants were rewatered after 14, 15 and 16 days' drought respectively, as indicated by arrows. For each line, *n *= 6. The data presented in Figures 3a and 3b were obtained from separate experiments.

**Figure 4:**
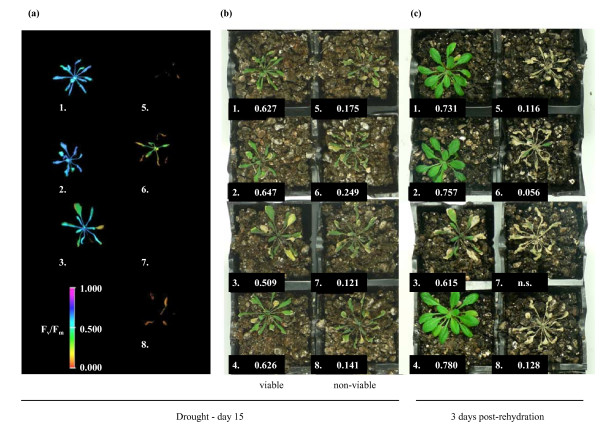
**Visual estimation of drought survival.** (a) False-colour representations of F_v_/F_m _measurements of Columbia plants following 15 days' drought treatment. The individual specimens were labeled 1 through 8, as indicated by the number below each plant. Note that false-colour images were not generated at F_v_/F_m _values of < ~0.125; for details, refer to Experimental Procedures. The image of plant #4 has been omitted for provision of the false-colour scale, however its F_v_/F_m _measurements were comparable to those of plant #1. (b) Photograph of the plants shown in (a). F_v_/F_m _measurements obtained from each plant are shown in the lower left corner of each punnet. The average F_v_/F_m _of control plants (not shown) was 0.800, providing a threshold F_v_/F_m _of 0.264. The 4 plants in the left column were classified as viable by application of the threshold test (F_v_/F_m _> 0.264), while the 4 plants in the right column were classified as non-viable (F_v_/F_m _< 0.264). (c) Photograph of the same 8 plants after watering was resumed for 3 days; n.s. = no signal detected.

To determine the threshold for viability, drought-treated Columbia, Landsberg and C24 plants exhibiting F_v_/F_m _measurements in the range 0.100–0.750 were rehydrated. None of the plants whose F_v_/F_m _measurements were less than the 33% of the mean F_v_/F_m _of watered control plants showed signs of recovery after 3 days, whereas the large majority (87%) of plants with F_v_/F_m _values above this threshold recovered following rehydration (Figure 3a, b). This visible recovery post-rehydration correlated with a gradual recovery in F_v_/F_m _(Figure 3b). For plants that showed no visible signs of recovery, F_v_/F_m _levels remained below 0.300. Thus, a threshold of 33% of the mean F_v_/F_m _of control plants provides a method to reliably identify non-viable specimens within a severely drought-affected population. The F_v_/F_m _threshold test provides a level of accuracy not possible through visual evaluation alone, as demonstrated in Figure 4. In this example, F_v_/F_m _measurements were performed on a subset of plants, all of which were classified visually as being dead (Figure 4a, b) despite the presence of viable specimens. Application of the threshold test correctly distinguished between the viable and non-viable plants, as confirmed through rehydration (Figure 4c).

### Case study: Measuring drought survival of water deficit-tolerant *Arabidopsis *mutants

**Figure 5:**
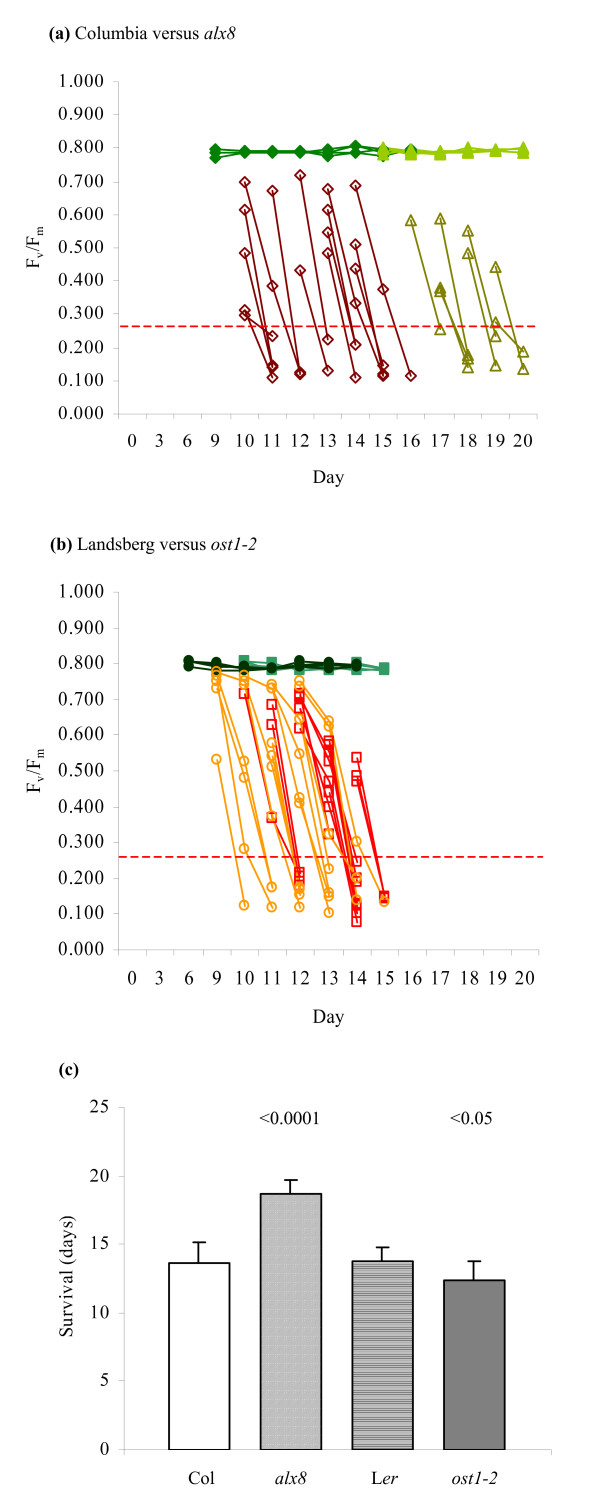
**Drought survival analysis of *alx8 *and *ost1-2 *plants.** (a, b) Application of the threshold test. The F_v_/F_m _measurements of individual (a) Columbia (◇) and *alx8 *(Δ), and (b) Landsberg (□) and *ost1-2 *(○) specimens are shown. Filled symbols represent controls; empty symbols represent plants that failed to evidence signs of recovery within 3 days of rehydration. The 33% threshold for a typical average control F_v_/F_m _of 0.800 is shown as a dotted line. For control populations, *n *= 4 for each line; for drought-treated populations, *n *= 15 for Columbia, Landsberg and *ost1-2*, and *n *= 8 for *alx8*. (c) Comparison of drought survival times of *alx8*, *ost1-2 *and wild-type plants. Error bars indicate standard deviation. Pairwise *t*-tests were performed for the mutant lines against survival times of their corresponding wild-type (Columbia for *alx8*, Landsberg for *ost1-2*), yielding *p*-values as shown.

To further appraise the precision of the threshold test for viability, it was utilized to perform an assessment of the survival during drought of an established water deficit-tolerant mutant, *altered APX2 expression 8 *(*alx8; *At5g63980) [42], and a drought-sensitive mutant, *open stomata 1–2 *(*ost1-2*; At4g33950) [43]. Monitoring of F_v_/F_m _levels and application of the threshold test (Figure 5a, b) permitted estimation of plant survival to a specific day (Figure 5c), with loss of viability confirmed via rehydration (data not shown). The experiment demonstrated that *alx8 *survived an average of 5.0 days longer than Columbia (*p *< 0.0001), while *ost1-2 *plants lost viability 1.4 days earlier than the Landsberg *erecta *wild-type parent (*p *< 0.05).

### Case study: Measuring drought survival of photosynthetically-impaired *Arabidopsis *mutants

**Figure 6:**
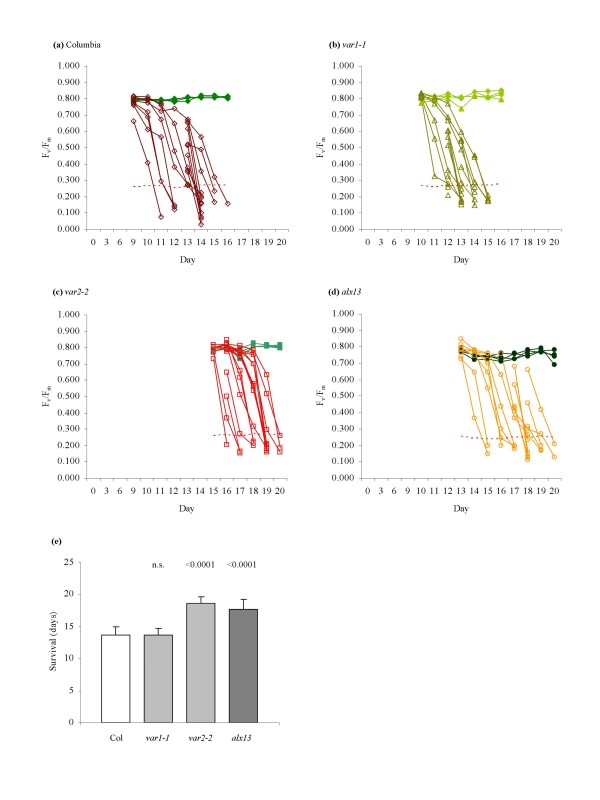
**Drought survival analysis of variegated lines of *Arabidopsis*.** (a-d) Application of the threshold test. The F_v_/F_m _measurements of individual (a) Columbia (◇), (b) *var1-1 *(Δ), (c) *var2-2 *(□) and (d) *alx13 *(○) specimens immediately prior to rehydration are shown. Filled symbols represent controls; empty symbols represent plants that failed to evidence signs of recovery within 3 days of rehydration. The 33% threshold for each line is shown as a dotted line. For control populations, *n *= 7 for each line; for drought-treated populations, *n *= 16 for each line. For clarity, only measurements from 4 control plants are shown. (e) Comparison of drought survival times of variegated lines. Error bars indicate standard deviation. Pairwise *t*-tests were performed against survival times of wild-type Columbia plants, yielding *p*-values as shown; n.s. = not significant. Data shown are the combined results of two separate experiments.

The use of the threshold test had now been validated on the common Columbia and Landsberg *erecta *ecotypes and on mutant plants with altered drought characteristics but comparable photosynthetic efficiencies. To determine whether the 33% F_v_/F_m _threshold test remained a valid predictor of viability when applied to *Arabidopsis *mutants with impaired photosynthetic activities, the drought survival of three variegated lines of *Arabidopsis *was evaluated. The *yellow variegated 1*, (*var1-1*; At5g42270) [44], *yellow variegated 2 *(*var2-2*; At2g30950) [45] and *altered APX2 expression 13 *(*alx13*) lines exhibit chlorotic sectoring and depressed photosynthetic efficiencies. Depending on the severity of chlorosis, the F_v_/F_m _values of control plants from the three mutant lines varied from 0.650–0.800, corresponding to threshold values in the range of 0.215–0.264. The threshold test was applied using the lower threshold values obtained from the mutant controls rather than the threshold of the non-chlorotic Columbia wild-type (Figure 6a–d). In this manner, survival times were estimated as shown in Figure 6e, with all plants failing to recover following rehydration.

### Case study: Comparison of a traditional rehydration survival test and the F_v_/F_m _threshold test

The threshold test was next applied to assess the drought survival of transgenic plants altered in the expression of an abiotic stress response transcription factor. The protein encoded by the HL-responsive gene *zinc-finger of *Arabidopsis *10 *(*ZAT10*; At1g27730) has been shown to function as both a positive and negative regulator of a number of genes involved in the oxidative stress response and is implicated in the activation and suppression of several abiotic stress response pathways, including osmotic, heat and salinity stress [46]. However, overexpression of *ZAT10 *has been variously reported as either conferring a marked increase in drought resistance [47] or not affecting the drought response at all [46] when assessed using the traditional re-watering survival tests.

**Figure 7:**
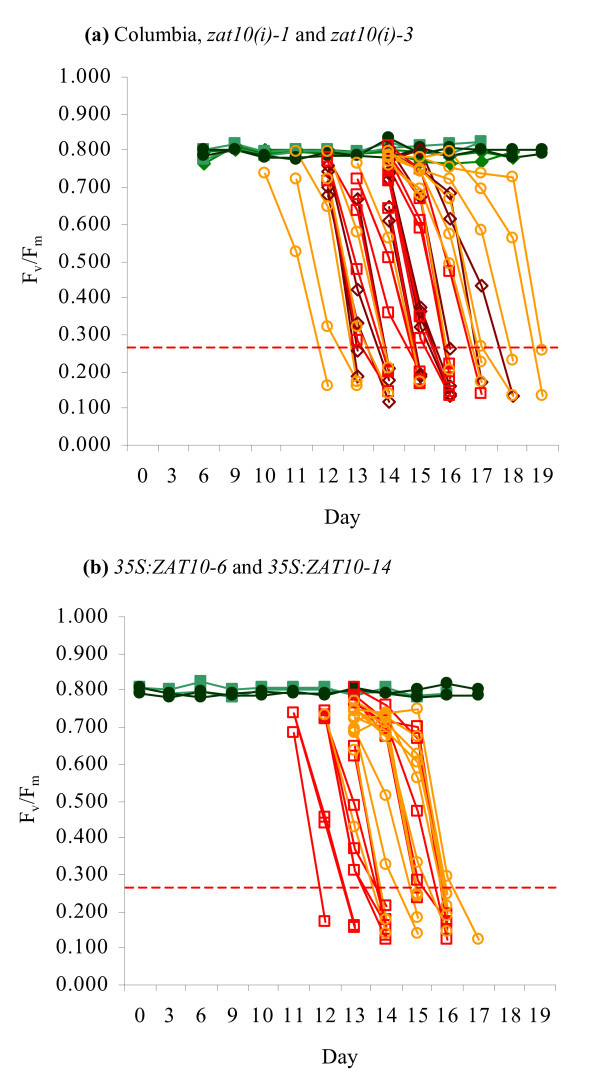
**Drought survival analysis of *ZAT10 *transgenic plants.** Application of the threshold test. The F_v_/F_m _measurements of individual (a) Columbia (◇), *zat10(i)-1 *(□) and *zat10(i)-3 *(○), and (b) *35S:ZAT10-6 *(□) and *35S:ZAT10-14 *(○) specimens are shown. Filled symbols represent controls; empty symbols represent plants that failed to evidence signs of recovery within 3 days of rehydration. The 33% threshold for a typical average control F_v_/F_m _of 0.800 is shown as a dotted line. For control populations, *n *= 2 for each line; for drought-treated populations, *n *= 13 for each line.

Two transgenic lines in which *ZAT10 *gene expression was suppressed via RNA interference (*zat10(i)-1 *and *zat10(i)-3*) and two lines in which *ZAT10 *was constitutively overexpressed under the direction of the cauliflower mosaic virus 35S promoter (*35S:ZAT10-6 *and *35S:ZAT10-14*) were subjected to drought survival analysis via both traditional rehydration methods and our threshold test [48]. As shown in Table 1a, in a traditional rehydration test three *zat10(i) *plants were shown to survive 20 days' drought treatment whereas all Columbia wild-type and *35S:ZAT10 *specimens had perished by this time. The inherent limitations of data obtained from this form of experiment make it difficult to draw substantive conclusions from these results as to whether this difference is significant and accurate. A threshold test survival experiment (Figure 7a, b), in comparison, indicated that length of survival in days was not statistically different for the two RNA interference lines and one of the overexpression lines (Table 1b). Only the *35S:ZAT10-14 *line displayed a significantly altered survival in comparison to the wild-type, a difference which may be considered negligible (*p*-value = 0.049).

**Table 1:** Drought survival analyses of *ZAT10 *transgenic plants using traditional and threshold test methods.

	Line	Col	*zat10(i)-1*	*zat10(i)-3*	*35S:ZAT10-6*	*35S:ZAT10-14*
(a)	# of viable plants post-rehydration	0/7	2/7	1/7	0/7	0/7

(b)	Survival time (days) ± s.d.	15.2 ± 1.5	15.3 ± 0.9	15.8 ± 2.5	15.5 ± 1.2	16.2 ± 1.1
	*t*-test	-	n.s.	n.s.	n.s.	~0.05

(a) Traditional rehydration survival test results. Plants were subjected to drought treatment for 20 days, after which watering was resumed; values indicate the number of plants that recovered within 3 days of rehydration. For each line, *n *= 7. (b) Threshold survival test results. For control populations, *n *= 2 for each line; for drought-treated populations, *n *= 13 for each line. s.d. = standard deviation. Pairwise *t*-tests were performed against survival times of wild-type Columbia plants, yielding *p*-values as shown; n.s. = not significant.

## Discussion

### Identification of a photosynthetic parameter suitable for assessment of drought progression

Here we have shown that F_v_/F_m _declines rapidly during late drought and can serve as an indicator of the latter phase of drought and subsequent loss of viability. Although it is possible that the other photosynthetic measurements obtained in this study could be employed as an indicator of viability, the F_v_/F_m _parameter is recommended for several reasons. First, as shown in Figure 1a, F_v_/F_m _values are typically very consistent between lines and individual plants; as such, any small decline is easily noticeable and signifies clearly that loss of viability is imminent. The consistency of the F_v_/F_m _parameter also increases the ease with which a threshold level can be defined. More importantly, unlike light-dependent parameters such as Φ_PSII _and NPQ, F_v_/F_m _is obtained from specimens in the dark-adapted state, negating the need for an extended period of illumination prior to measurement. Thus, as measurement of F_v_/F_m _can be completed using a single saturating pulse, rapid screening of a large number of plants may be achieved.

### Quantification of viability using chlorophyll fluorescence measurements

To employ the decline in F_v_/F_m _as a means of determining viability during drought, it was necessary to identify a threshold F_v_/F_m _level that would reflect a point at which recovery was no longer possible. As it is of course impossible to define an exact threshold level beyond which viability is lost, we identified a conservative threshold of 33% of control specimen measurements and showed that, in practice, decline of F_v_/F_m _below this level no plants were viable upon re-watering (Figure 3; Figure 4; Figure 5a, b; Figure 6a-d; Figure 7).

To validate the efficacy of the threshold test, the technique was employed to assess the drought performance of the *alx8 *and *ost1-2 *mutant lines previously identified as drought-resistant and drought-sensitive, respectively [42,43]. Using this method it was possible to monitor the viability of drought-affected plants and evaluate the survival times of individual plants in a precise and quantifiable manner (Figure 5). The robustness of the threshold test was further confirmed through its application in a drought survival analysis of three variegated lines of *Arabidopsis*. The variegated lines *var1-1*, *var2-2 *and *alx13 *are sensitive to photoinhibitory damage and consequently have impaired photosynthetic efficiencies. This impairment is manifest in reduced F_v_/F_m _levels in each of the three mutant lines, which in turn necessitated the application of their respective control F_v_/F_m _levels to calculate the 33% thresholds. The threshold test successfully ascertained loss of viability in specimens of all three mutants, demonstrating its utility even in situations where photodamage and differing photosynthetic capacities are present (Figure 6). Intriguingly, the test also indicated differences in drought survival between the mutants and wild-type, a discovery that is under further investigation.

As a demonstration of the advantages of the threshold test, the drought survival of *ZAT10 *transgenic lines were evaluated using both the threshold test technique and the traditional rehydration method. The limitations of the traditional rehydration test (Table 1a) are apparent: although three *zat10(i) *specimens remained viable at the end of the experiment, the extent of this increased survival is cannot be established as there is no indication of the time at which wild-type plants became inviable. Indeed, as this test does not yield survival data for individual specimens it is not possible to determine whether the surviving plants are outliers among their populations, nor can the variability in survival times within each population be estimated. It cannot be ascertained at all whether *35S:ZAT10 *plants exhibit altered drought survival compared to the wild-type.

The threshold test, in contrast, provides a far more informative assessment of drought survival. From the data presented in Table 1b and Figure 7 it is immediately evident that the survival times of all of the lines in the threshold test experiment were very similar, with average survival times indicating that the loss of viability of all lines occurred within a 1-day period. Statistical assessment of the survival times of the transgenic lines indicated that *35S:ZAT10-14 *plants may remain viable during drought for slightly longer than the wild-type, but also show that any increased viability is at most marginally significant. Note that the results shown in Table 1 are for the purposes of demonstrating differences in the interpretation of traditional and threshold survival test methods and do not represent a comprehensive analysis of the effect of altered *ZAT10 *expression on the drought response; such an investigation would require monitoring of additional parameters such as the extent of *ZAT10 *overexpression/suppression.

### Applications and suggestions for using the threshold test for measuring viability

The threshold test offers a reliable, rapid and quantitative alternative to conventional studies of drought survival in *Arabidopsis*. As only minimal technical expertise and a basic understanding of chlorophyll fluorometry are required to obtain the necessary measurements, the threshold test may appeal to a broad spectrum of plant science laboratories. Further, this procedure does not require the use of expensive or esoteric equipment. Although the results presented in this analysis were produced using an IMAGING-PAM system (Walz; Effeltrich, Germany) and have also been validated using a Chlorophyll Fluorescence Imager (Technologica; Colchester, UK) (data not shown), a number of less costly devices are available. For example, the FluorPen (Photon Systems Instruments; Brno, Czech Republic) and Pocket PEA Chlorophyll Fluorimeter (Hansatech; Norfolk, UK) offer convenient means of monitoring F_v_/F_m _levels both in the laboratory and in the field at low cost. Instruments such as these are also amenable for determination of the OJIP fluorescence transient [29], and therefore offer the potential for assessment of plant performance during early and moderate phases of drought treatment [32] in addition to drought survivability. However, when employing a fluorescence probe it may be necessary to acquire several measurements in order to account for heterogeneities in photosynthetic efficiencies across the leaf surface of plants, particularly severely drought-stressed specimens.

While beyond the scope of this report, it is easily conceivable that the threshold test may be successfully applied to monitor the survival of plants under different forms of abiotic stress, particularly those that cause progressive deterioration of photosynthetic efficiencies. Prolonged cold or light stress, for example, can induce accumulative photoinhibitory damage to the photosynthetic machinery to the point at which a specimen is no longer able to sustain vital functions [49,50]. Likewise, it is quite likely that the threshold test could be adapted for use with other plant species. We have targeted *Arabidopsis *as this rapid test could be applied to mutant and genotype screens in advance of detailed analyses of water relations.

### A discussion of the drought-induced changes in chlorophyll fluorescence parameters in *Arabidopsis*

While an investigation of the physiological and photochemical bases of the observed drought-induced changes in chlorophyll fluorescence was not an objective of this report, they will be discussed briefly in this section. Measurements of the maximum and operating efficiencies of PSII, as represented by F_v_/F_m _and Φ_PSII _respectively, indicated that there was no significant perturbation of PSII photochemistry or electron transport capacity within the photosystems despite the initial significant decreases in RWC (Figure 1a, b; Figure 2). Indeed, only when plant water reserves declined to critical levels (<20% RWC) were F_v_/F_m _measurements consistently reduced. These results are similar to observations in sunflower, where F_v_/F_m _was unchanged across a comparable range of water deficit stress [22], in pea, where only a slight decrease was noted despite RWC as low as 20% [51], and in triticale, where extended drought failed to alter F_v_/F_m _significantly [52]. Thus, although drought is known to cause gradual inhibition of assimilatory photochemistry through both stomatal [24] and metabolic [22] restriction of CO_2 _availability, photosynthetic electron transport may be maintained throughout the course of drought treatment through dissipation of excess excitation energy by alternative electron sinks [40,53].

During prolonged water deficit, severe reduction of cellular water content results in enhanced leaf senescence, as reflected by elevated levels of reactive oxygen intermediates and chlorophyll degradation [54,55]. Thus, it is possible that the rapid decline in photosynthetic parameters observed during the latter stages of drought is attributable to senescence-induced chlorosis and disruption of the photosynthetic apparatus. The rapid photosynthetic decline during late drought may therefore be a consequence of the damage to PSII reaction centres or associated chlorophylls [56], although it has previously been suggested that drought-induced suppression of photosynthetic efficiencies may be due to the deterioration of an electron carrier at the donor side of PSII, rather than destruction of the PSII reaction centre or of chlorophyll molecules [57]. Chlorophyll fluorescence measurements may also be influenced by non-photosynthetic alterations in leaf physiology associated with prolonged drought, such as changes in leaf angle due to loss of turgor. Irrespective of the mechanisms responsible for the observed photosynthetic decline, though, the rapid change in the F_v_/F_m _parameter may nonetheless be employed via the threshold test as a means of estimating drought survival.

## Conclusion

In this report, we describe a method of determining the survival of drought-treated *Arabidopsis *utilizing measurements of the F_v_/F_m _chlorophyll fluorescence parameter. Although photosynthetic parameters remained mostly unchanged during the first phase of drought treatment, a sudden deterioration in photosynthesis was observed to occur just prior to the terminal stages of drought and the loss of plant viability. By correlating the decline in the F_v_/F_m _parameter to this loss of viability, a procedure was developed to allow the monitoring of survival under water deficit conditions, namely defining a threshold of 33% of well-watered F_v_/F_m _values. The versatility of this technique was demonstrated through comparison of the drought performance of a number of *Arabidopsis *cultivars and to a variety of mutants with altered drought tolerance or photosynthetic capacity. As a rapid, non-invasive and inexpensive procedure, the threshold test for survival holds much value in screening for altered responses to drought in *Arabidopsis *germplasm. This procedure may complement existing methods of evaluating drought performance utilizing chlorophyll fluorescence [32], and increase the number of tools available for assessment of this and potentially other plant stresses.

## Methods

### Plant growth conditions and drought treatments

All *Arabidopsis thaliana *plants were cultivated under 100 ± 10 μmol photons m^-2 ^s^-1^, 8-hour photoperiod, 23°C/22°C day/night temperatures, 50%/70% day/night humidity. Seed were sown on a moistened, loosely-packed 3:1 mixture of soil:vermiculite, then vernalized at 4°C in darkness for 72 hours before transfer to growth conditions. Prior to initiating drought treatment plants were watered every second day, with every third watering supplemented with 0.5× Hoagland's Fertilizer [58]. After 7 days' growth, seedlings were thinned to leave one plant per punnet. Drought treatments were initiated when plants were 28 days' old, at which time all specimens were at ~12-leaf stage, with the exception of assays involving variegated mutant lines and *ZAT10 *transgenic lines. In the experiments involving variegated mutants, in order to account for differences in developmental rates, Columbia populations began treatment at 30 days of age, *alx13 *at 33 days, and *var1-1 *and *var2-2 *at 37–40 days, at which times all plants were at ~14-leaf stage. For the traditional rehydration survival analysis of *ZAT10 *transgenic lines, drought treatment was initiated at 28 days' of age; after 20 days rehydration was performed as described below. For the threshold survival test of *ZAT10 *lines, drought treatment was initiated at 42 days' of age.

For drought treatments, all plants were first provided with a sufficiency of water. Punnets containing plants to be subjected to drought were then removed to water-free trays with spaces between specimens to allow air flow, and further watering withheld; all other environmental conditions were maintained as described above. Control plants remained under watered conditions for the duration of the experiment. Where rehydration was necessary, punnets were returned to watered trays for 72 hours. Plants that failed to exhibit any physical signs of recovery within this time were deemed to have lost viability.

### Measurement of photosynthetic parameters

Chlorophyll fluorescence measurements were performed using an IMAGING-PAM chlorophyll fluorometer and ImagingWin software application (Walz; Effeltrich, Germany). For assessment of dark- and light-adapted parameters, a dark-light induction curve was performed. Dark-adapted plants were subjected to an initial saturating pulse of >1800 μmol photons m^-2 ^s^-1^, followed by a 40" delay in darkness and subsequently 10' of actinic illumination with saturating flashes at 20" intervals. An actinic irradiance of 100 ± 10 μmol photons m^-2 ^s^-1 ^was used to simulate growth conditions. The following parameters were derived from the final measurements obtained after the 10' light adaptation: Φ_PSII_, Φ_NO_, NPQ and ETR. F_v_/F_m _values were taken as the measurement of Φ_PSII _at time zero. The four primary fluorescence signals – F_o_, F_m_, F_s_' and F_m_' – from which the above photosynthetic parameters were derived are shown in Additional file 2. For background information regarding photosynthetic parameters and theoretical aspects of chlorophyll fluorescence, refer to [25,26,39,41]. To account for variations in photosynthetic parameters across the surface of individual plants, the data presented are the average values obtained across individual rosettes. Note that, where false-colour images of the F_v_/F_m _parameter are shown, the ImagingWin software eliminates pixels in areas where F_m_<0.040 in order to reduce background noise. For this reason, F_v_/F_m _images of certain severely drought-affected plants were unobtainable; in these instances the average F_v_/F_m _measurements alone are presented.

For experiments requiring only determination of F_v_/F_m_, measurements were obtained from application of a single saturating pulse to dark-adapted plants. All photosynthetic measurements were performed prior to dawn, after 12–16 hours' dark adaptation. For accurate measurement of F_v_/F_m _a dark adaptation of >15 minutes is typically sufficient.

### Determination of relative water content

For measurements of rosette RWC, the entire aerial parts of the plant were harvested using a single incision to the base of the stem, and the fresh weight (FW) of the rosette determined. The rosette was then floated on distilled water in darkness at 4°C for 24 hours before determination of turgid weight (TW). The rosette was then placed in a paper envelope and dried at 65°C for 24 hours, and the desiccated sample weighed once more to determine dry weight (DW). RWC was calculated from these measurements as follows:

RWC = ((FW-DW)/(TW-DW)) × 100%

## Competing interests

The authors declare that they have no competing interests.

## Authors' contributions

NW conceived of the described procedure, performed all photosynthetic measurements and drought studies and prepared the manuscript. All authors participated in experimental design and data analysis. All authors read and approved the final manuscript.

## Supplementary Material

Additional File 1 **Additional measurements of photosynthetic parameters during progression of drought.** Click here for file

Additional File 2 **Raw chlorophyll fluorescence parameters (F_o_, F_m_, F_s_' and F_m_') during progression of drought.** Click here for file
