# Supplementary material for: A rapid, non-invasive procedure for quantitative assessment of drought survival using chlorophyll fluorescence
Source: Plant Methods. 2008 Nov 11;4:27. doi: 10.1186/1746-4811-4-27 (PMC2628343; doi:10.1186/1746-4811-4-27)
Supplement: Additional File 1 — Additional measurements of photosynthetic parameters during progression of drought. [file 1746-4811-4-27-S1.ppt]

## Slide 1
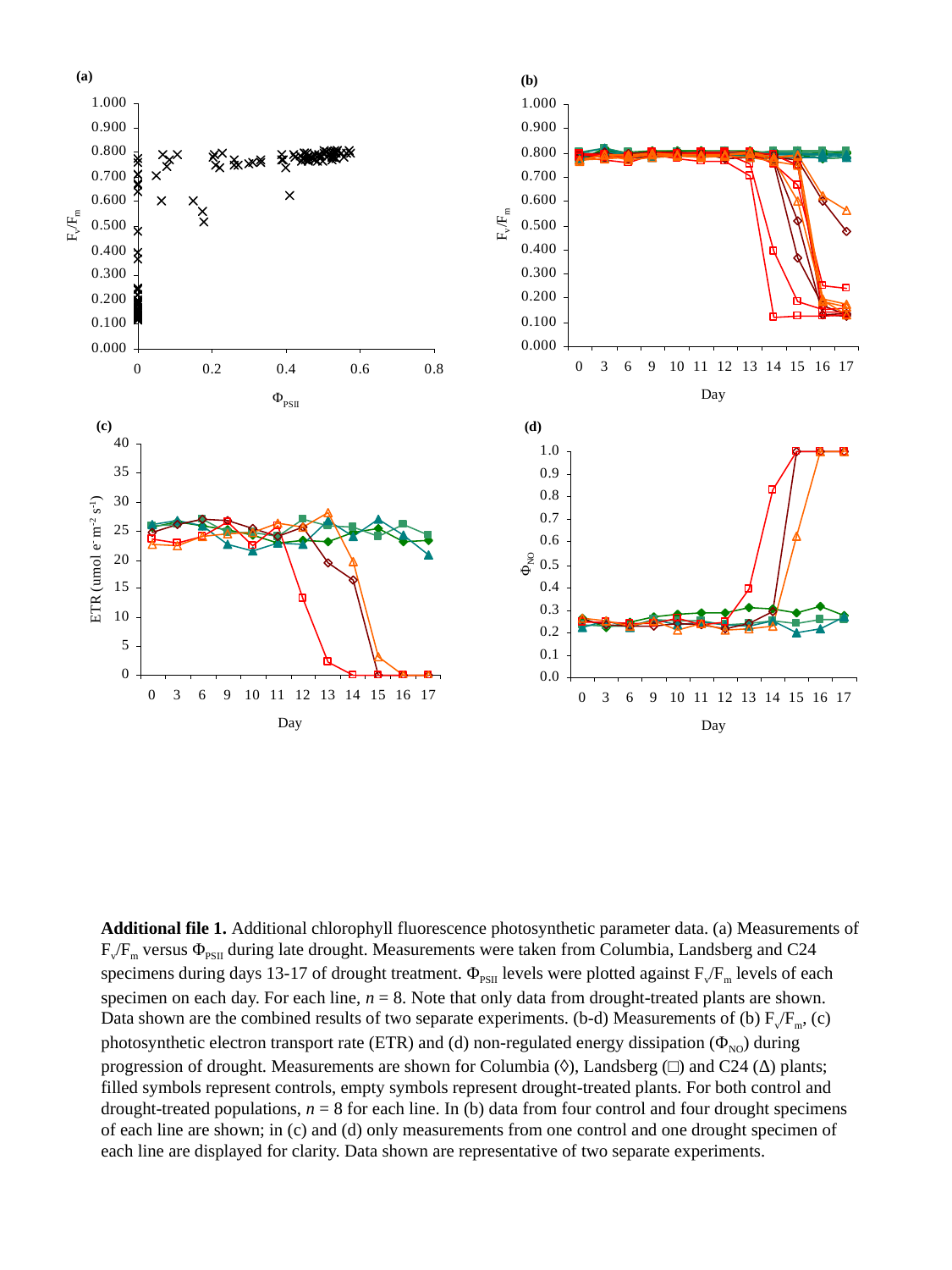

(b)
(a)
(c)
(d)
Additional file 1. Additional chlorophyll fluorescence photosynthetic parameter data. (a) Measurements of Fv/Fm versus ΦPSII during late drought. Measurements were taken from Columbia, Landsberg and C24 specimens during days 13-17 of drought treatment. ΦPSII levels were plotted against Fv/Fm levels of each specimen on each day. For each line, n = 8. Note that only data from drought-treated plants are shown. Data shown are the combined results of two separate experiments. (b-d) Measurements of (b) Fv/Fm, (c) photosynthetic electron transport rate (ETR) and (d) non-regulated energy dissipation (ΦNO) during progression of drought. Measurements are shown for Columbia (◊), Landsberg (□) and C24 (Δ) plants; filled symbols represent controls, empty symbols represent drought-treated plants. For both control and drought-treated populations, n = 8 for each line. In (b) data from four control and four drought specimens of each line are shown; in (c) and (d) only measurements from one control and one drought specimen of each line are displayed for clarity. Data shown are representative of two separate experiments.
