# Supplementary material for: A rapid, non-invasive procedure for quantitative assessment of drought survival using chlorophyll fluorescence
Source: Plant Methods. 2008 Nov 11;4:27. doi: 10.1186/1746-4811-4-27 (PMC2628343; doi:10.1186/1746-4811-4-27)
Supplement: Additional File 2 — Raw chlorophyll fluorescence parameters (Fo, Fm, Fs' and Fm') during progression of drought. [file 1746-4811-4-27-S2.ppt]

## Slide 1
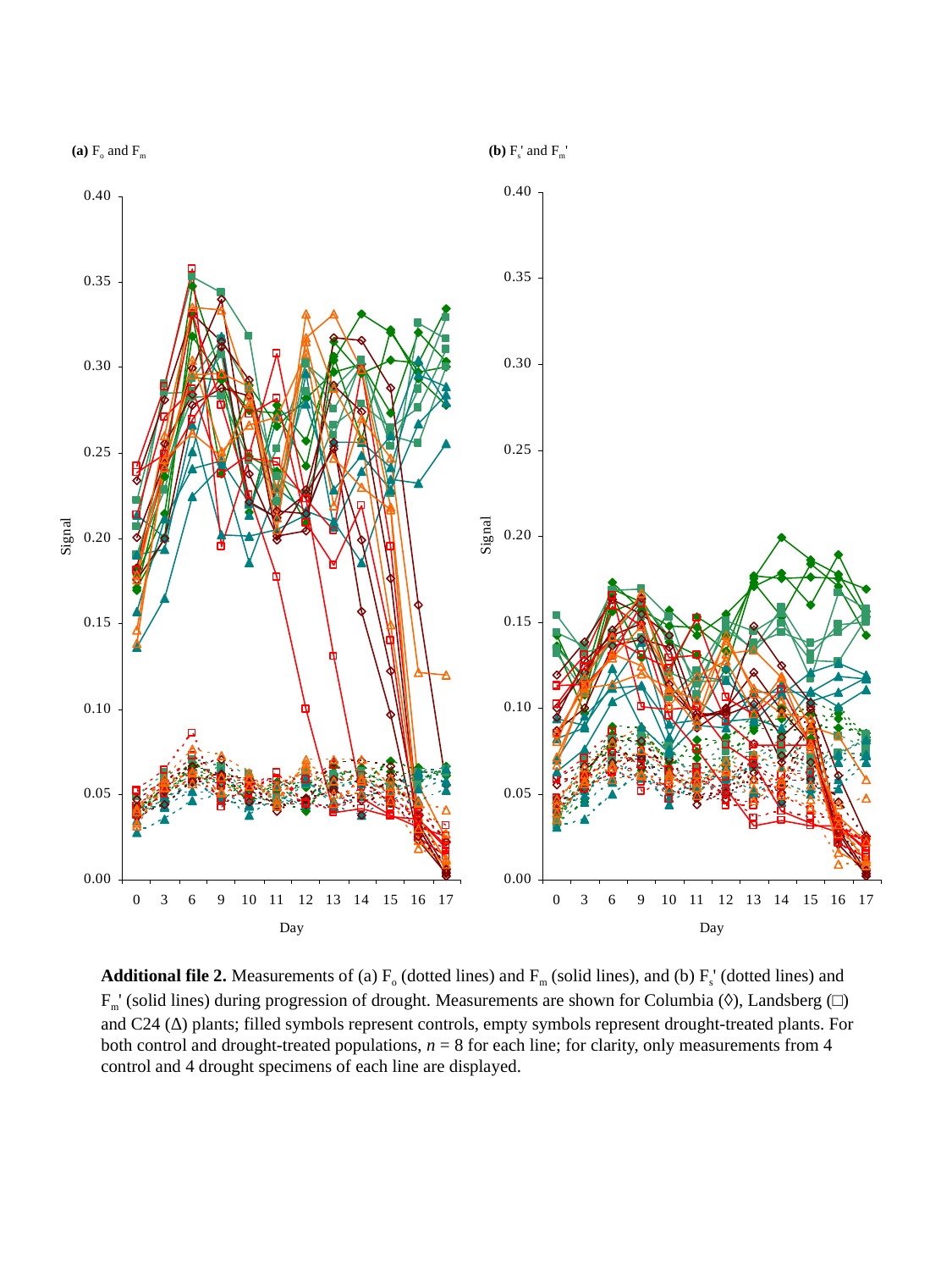

(a) Fo and Fm
(b) Fs' and Fm'
Additional file 2. Measurements of (a) Fo (dotted lines) and Fm (solid lines), and (b) Fs' (dotted lines) and Fm' (solid lines) during progression of drought. Measurements are shown for Columbia (◊), Landsberg (□) and C24 (Δ) plants; filled symbols represent controls, empty symbols represent drought-treated plants. For both control and drought-treated populations, n = 8 for each line; for clarity, only measurements from 4 control and 4 drought specimens of each line are displayed.
